# Exploring mental health problems and support needs among pregnant and parenting teenagers in rural areas Of Limpopo, South Africa

**DOI:** 10.1186/s12905-024-03040-z

**Published:** 2024-04-13

**Authors:** Livhuwani Muthelo, Masenyani Oupa Mbombi, Peter Mphekgwana, Linneth Nkateko Mabila, Inos Dhau, Joseph Tlouyamma, Reneilwe Given Mashaba, Katlego Mothapo, Cairo Bruce Ntimane, Kagiso Peace Seakamela, Rathani Nemuramba, Eric Maimela, Tholene Sodi

**Affiliations:** 1 Department of Nursing Science, University of Limpopo, SOVENGA, Private Bag X1106, Polokwane, 0727 South Africa; 2 Research Administration and Development, University of Limpopo, SOVENGA, Private Bag X1106, Polokwane, 0727 South Africa; 3 Department of Pharmacy, University of Limpopo, SOVENGA, Private Bag X1106, Polokwane, 0727 South Africa; 4 Department of Geography and Environmental Studies, University of Limpopo, SOVENGA, Private Bag X1106, Polokwane, 0727 South Africa; 5 Department of Computer Science, University of Limpopo, SOVENGA, Private Bag X1106, Polokwane, 0727 South Africa; 6 DIMAMO Population Health Research Centre, University of Limpopo, SOVENGA, Private Bag X1106, Polokwane, 0727 South Africa; 7 Department of Public Health, University of Limpopo, Private Bag X1106, SOVENGA 0727, Polokwane, South Africa; 8 Research Chair Mental Health, University of Limpopo, Private Bag X1106, Polokwane, 0727 SOVENGA South Africa

**Keywords:** Mental health, Spousal support, Stigmatisation, Teenage pregnancy, Depression

## Abstract

**Background:**

Globally, teenage pregnancy is among the most social problems, affecting 21 million adolescents aged 15–19. Due to the increased responsibility of prenatal and postnatal care for their infants without support, pregnant and parenting teenagers, tend to experience mental health problems. Factors contributing to these problems among pregnant and parenting teenagers in rural African settings have hardly received scholarly attention and, therefore, are less understood. The purpose of the study was to explore mental health and challenges among pregnant and parenting teenagers,.

**Method(s):**

The study adopted a qualitative descriptive, exploratory, and phenomenological design. Purposive sampling was used to select 22 pregnant and parenting teenagers 18 years or older. Data were collected in ten selected clinics within the Demographic Surveillance Systems (HDSS) of DIMAMO and analysed using qualitative content analysis.

**Results:**

The findings reveal that pregnant and parenting teenagers in rural areas experience various mental challenges such as depression. These challenges are caused by social problems such as stigmatisation, lack of support from families and friends, as well as parenting demands that contribute to poor progress at school or dropouts. Pregnant teens expressed concerns about the lack of spousal support resulting from abandoning their partners.

**Conclusions:**

Stress and depression were self-reported as mental problems among participants with various psychosocial implications, such as school dropout and miscarriage. There are various contributing factors to the mental health problems identified among pregnant and parenting teenagers, including inadequate family and spousal support. Access to integrated reproductive, psychosocial, and mental health services could be essential for these pregnant and parenting teenagers, to improve their mental well-being and improve the support system.

## Introduction

Globally, 13 million of the 21 million teenage teenagers, who become pregnant each year between the ages of 15 and 19 give birth, and most of them are from low and middle-income countries (LMICs) [1, 2]. In sub-Saharan Africa, 35% of pregnancies among 15 to 19 years were unintended, unwanted, or mistimed, and most of the relationships during this developmental period were considered unstable. About two thirds of these unplanned pregnancies end in live births, and the other third ends in risky abortions [1–3]. Since teenage pregnancy occurs during the development period, adolescents are often forced to transition from childhood to adulthood, which can result in physical, biological, emotional, social and mental health challenges [4, 5]. During the transition process, pregnant adolescents are exposed to many health-related challenges that could negatively affect them and put their health and that of their unborn children at risk [5, 6]. In addition, stress and anxiety are frequent during pregnancy, particularly among unmarried adolescents, because adjusting to heightened expectations can be a period of emotional and mental strain [7–9].

Again, during the transition to adulthood, pregnant adolescents are more likely to find it difficult to deal with pregnancy-related problems. This may result in feelings of low self-esteem and various mental health problems such as stress, anxiety, fear, depression, and suicidal thoughts [10]. Scholars have argued that depressed and anxious mothers may develop strong feelings of annoyance, which could make it difficult for them to care for their children [11, 12]. Furthermore, several researchers have shown that teenage pregnancy and parenting are associated with an increased risk of problems during pregnancy and delivery, adverse neonatal outcomes with recurrent pregnancies, and developmental abnormalities in their children [10, 11, 13]. Consequently, the children tend to have negative emotions with decreased cognitive performance as young as two months. They may also have decreased cognitive performance, which is a negative consequence and a serious concern that could continue to affect them even during their college years [12, 14–16]. Studies have shown that pregnant and parenting teenagers, face additional challenges in life after giving birth, including lower educational attainment, lower incomes, and higher unemployment rates [17, 18]. Furthermore, parenting teenagers are at risk of experiencing psychiatric disorders and suicidal behaviour, with the prevalence of a higher mortality rate later in life. Research conducted in Soweto in South Africa (SA) indicates that young women between the ages of 13 and 16 had an abortion rate of 23%, while with teenagers between 17 and 19, the abortion rate was 14.9% [1, 19].

A group of parenting teenagers, in SA were found to have a much higher incidence of possible mental illnesses than their peers who had never given birth (18.2% vs. 9.6%) [12]. Both obstetric difficulties and poor postpartum mother functioning are associated with mental disorders. These circumstances can adversely affect maternal mental health, parenting, and behavioural outcomes for their children. It is against this background that the research team aimed to explore mental health problems, contributing factors of mental health, consequences of mental problems, and support needs among pregnant teenagers residing in selected rural communities of Limpopo Province.

## Material and methods

### Study Design

The qualitative exploratory, descriptive, and phenomenological design was adopted. This study design uses a flexible approach that produces high-quality data that enable a deeper understanding of lived experiences [20]. About the support and mental health challenges experienced by pregnant and parenting teenagers, in selected rural communities of Limpopo province.

### Study site

The study was carried out in ten clinics within the DIMAMO Population Health Research Centre (PHRC), which is one of the health and demographic surveillance sites affiliated with the South African Population Research Infrastructure Network (SAPRIN), which is hosted by the South African Medical Research Council and funded by the National Department of Science and Innovation (DSI). The site is located in the Capricorn District of Limpopo Province and covers a population of approximately 100,000 people. At the ten selected clinics, maternal and child health services are provided to women during and after pregnancy.

### Population and sampling

In the context of this study, most of the participants resided in rural, underprivileged communities ravaged by poverty. The target population of the study was all pregnant and parenting teenagers, aged 15 to 19 years or under. Purposive sampling was used to select 22 participants with lived experiences of mental health problems during their adolescent pregnancy and early parenting. The selection criteria of the participants were being a teenage woman, being pregnant, including being a teenage mother. All participants were enrolled for antenatal care (ANC) or child growth monitoring in the selected clinics.

### Data collection

Individual in-depth interviews were conducted with participants between the period of September 2022 to November 2022, who were recruited through the clinic managers. The project team explained the purpose and objectives of the study to managers and staff, who then identified the pregnant and parenting teenagers, who were enrolled as participants in the study. The research team then introduced itself to them and explained the purpose of the study. Arrangements for the date and time for data collection were made. Interviews were carried out in private rooms within the clinic premises by LM (Female) and MOM (Male), who are experienced qualitative researchers, MOM, RGM, CBN, KM, and RN. The interviews were conducted following the interview guide with questions asked. The researchers used audio recorders along with notepads to record the interviews that lasted approximately 45 min. Data were collected until data saturation was reached at participant number twenty-two. The sociodemographic profiles of the participants are detailed elsewhere [21].

### Data analysis

Qualitative content analysis was used as recommended by Creswell and Guetterman [20] to analyse the data. A team of three authors organised the data, including the transcription of all recorded audio and filed notes. Three authors read through the transcripts to obtain a general sense of the material. Thereafter coding of data was done, which includes locating segments to assign code labels. The codes were examined to build the themes and their descriptions. A meeting was arranged with other authors to identify and refine the themes. Reporting the themes as qualitative findings. Finally, all the authors agreed on the themes and subthemes outlined in Table 1. The adoption of content analysis assisted the research team in interpreting the content of text data through the methodical coding classification process. The research team allowed these themes and subthemes to flow from data through intense immersion in the data to allow new insights to emerge. The themes and subthemes are presented in Table 1.

**Table 1:** Themes and sub-themes reflecting mental health problems and support needs among pregnant and parenting adolescents in selected rural clinics of Limpopo Province

Themes	Sub-themes
Contributory factors to mental health problems among pregnant and parenting teenagers,	Mixed perceptions about mental illness among pregnant teens Stigmatisation by the community and friends Being a single parent is challenging versus lack of partner support
Psychosocial problems associated with adolescent pregnancy	Self-actualisation challenges School dropout Miscarriage and Abortion
Diverse support systems and roles to minimise mental problems pregnant and parenting teenagers,	Mixed perceptions about the role of the family, partners and community as a support system Role of the Healthcare Workers / Clinics Support System Role of culture among pregnant and parenting teenagers, School and social worker role as a support system

## Results

### Contributory factors to mental problems among pregnant and parenting teenagers

The study findings illustrated the contributing factors to mental problems among pregnant and parenting teenagers, resulting in the following themes.

#### Mixed perceptions of mental illness among pregnant and parenting adolescents

We observed mixed perceptions about mental illness in rural areas, with some pregnant and parenting adolescents attributing mental illness to worries about too many things, especially when one has problems with family and friends. The adolescents argued that they experienced depression and stress during their early motherhood state. The following assertions illustrate these findings:

Ok, depression is when someone is stressed because you are pregnant, for example, like you, you are stressed at school or home because you are pregnant (Participant 10).

#### Community and friend stigmatisation

The study findings suggest that stigmatisation by the community and friends is a contributing factor to depression among pregnant and parenting adolescents in rural areas. The teenagers expressed fear of being judged and not having support from family and friends, who tend to discriminate against them due to their pregnancy. The following extracts illustrate this.

Fear of being judged and not having support or anyone to talk to… If you come from a family that does not support your friends who gossip about you and the community that also gossips about you, ‘stopped to overthink” (Participant 4).

#### Being a single parent is challenging versus lack of partner support

Pregnant and parenting teenagers expressed concern about being a single parent. They perceived this as challenging, especially when there was a lack of spousal support. Concern about the father who disappears when needed or the father abandoning the child was emphasised. This is reflected in the following quotes.

On the other hand, the father of the child has abandoned his child. As a mother, I started to feel stressed about food and clothing for both me and the child (Participant 11).

### Psychosocial problems due to adolescent pregnancy

The study found that some psychosocial problems arise from teenage pregnancy. These included self-actualisation challenges, school dropout, miscarriage, and abortion. The following themes support the objective:

#### School dropout

In addition to poor academic performance, pregnant and parenting teenagers expressed the desire to drop out of school. The reason for this is the absence of guardians or babysitters for their newborns. These findings are supported by the following quotations.

We end up dropping out of school because we have people to babysit for us, and also when you are with your friends who have not had children, you feel like you are no longer part of them (Participant 2).

#### Self-actualization challenges

The findings indicate that the participants tend to fail to reach self-actualisation due to teenage pregnancy. They argued that they might not achieve their goals or dreams due to the disruption of their academic performance at school. The following quotes support the findings:

…I thought of running away from home because I thought they would be better off without me because I thought I was the problem (Participant 3).

#### Miscarriage and abortion

Other pregnant teenagers wish to abort their babies due to a perceived lack of support systems. Some of them were concerned about the possibility of a miscarriage due to excessive worries about their status. These findings are supported by the following quotations.

You can lose your baby by miscarriage, or your baby’s condition may not be good when you give birth… When a pregnant woman has a lot on her mind, it affects baby growth and she will give birth to a premature baby or a disabled baby. You might end up stressed out because your classmates talk about it (Participant 1).

### Availability of support systems and their role in minimising mental problems among pregnant teenagers

The study findings demonstrated that the availability of support systems helps minimise mental problems among pregnant teenagers. They felt that family, school, healthcare facilities and community played a significant supportive role. The following themes demonstrate how we achieved the objective:

#### Role of family and community as support systems

The study findings demonstrated mixed perceptions about the role of family and community as support systems among pregnant and parenting adolescents. They acknowledged the role played by family members and the support received at home. As reflected in the following quotes, they felt that family, friends, and neighbours provided emotional support.

The people we stay with at home support us… Just for them to always talk about pregnancy and pregnancy-related issues and for community members to stop pointing fingers and gossiping about us (Participant 1).

#### Role of healthcare workers and clinic support systems

The pregnant teens felt that healthcare workers in clinics play an important role in their mental well-being. They found the support groups there helpful. The following quotes reflect this sentiment:

…Yes, clinics support teenage moms and pregnant teens - Yes, at the clinic, we have social workers. Therefore, you can get support from them Participant 8).

#### The role of culture

Teens pregnant and parenting were asked to share their views on the role of culture in their lives. They were of the view that culture plays a significant role in guiding, as shown in the following quotes:

It teaches us what kind of clothes to wear and not wear, saying that your belly does not have to show/ or the next person must not see how big your tummy is. However, it has no clear role in depression (Participant 1).

They help us to be informed of how things were done in the past e.g. consulting with your ancestors before the baby is born and therefore support us accordingly (Participant 2).

#### The supportive role of teachers and social workers

The findings of the study illustrated the role of teachers and social workers as valuable support systems for pregnant and parenting adolescents. The following quotations lend support to this finding.

…Yes, social workers are there to help us, but not the community. A Centre for Women and Pregnant Teenagers is opened to help and motivate teens with food parcels now and then (Participant 11).

## Discussion

The reported contributing factors to mental health problems among pregnant and parenting teenagersinclude inadequate support systems from partners, family, and the community, and being a single mother without financial support. It is noted that maternal and prenatal health is a concern among adolescents [22]. The findings reveal that these teenagers experience mental problems, specifically depression and stress, as most of them find themselves worrying about many things during their pregnancy or parenting journey. This is consistent with the findings of other studies that pregnancy poses a serious challenge to the mental health of pregnant and parenting adolescents [22–25]. In addition, adolescents indicated that the contributing factors to most of their worries include problems with their families and friends related to support and acceptance of their pregnancy. Stigmatisation from the community and friends was also described as a contributing factor to depression. The literature denotes that pregnant and parenting adolescents are stigmatised by society and this negatively impacts their willingness to seek health services [22, 26–28]. Therefore, it is important to raise awareness in the community and educate them about the importance of social support for pregnant and parenting teenagers from their families, the father of the baby, friends, and the community to kerb the detrimental effects of depression among this group.

Pregnant teenagers expressed their concern about single parenthood, which is challenging, especially lack of spousal support resulting from abandonment of the pregnancy. This experience is similar to that of teenage mothers in a study by Ntshayintshayi et al. [25], who stated that parenting is hard and stressful, and therefore that parenting needs spousal support [29]. The challenges associated with teenage pregnancy and parenting affect emotions and end in somatization, with signs and symptoms that would be harmful to healthy pregnancy or child [24, 30]. Pregnancy risks are related, among others, to psychological aspects and lack of family support, which may lead to high stress levels. In some families, teen pregnancy may be considered natural when there is a stable union between the teenager and the parents [31]. Family support is described as a fundamental need for parenting teenagers. Grandmothers are considered an important foundation of support in this regard, as they have numerous roles in the family, including advising, coaching and coordinating activities of other family members, and managing family resources [32, 33]. Contrary to the findings [25] that demonstrated that nurses have a negative attitude toward teenage pregnancy, the participants revealed that nurses did not treat them well during clinic visits. This experience made participants’ lives difficult.

The observed support systems for pregnant teenagers include various family support systems, spousal and community support systems, and support from school and social workers in clinics. In particular, adolescents experience these support systems differently, with some satisfied and others not satisfied. Multidisciplinary healthcare is considered an essential need for adolescent mothers and their children. Holistic healthcare that combines medical and psychosocial services is necessary for teenagers. Pregnant teenagers and adolescent mothers need access to medical and psychosocial support. Support services are crucial in addressing mental health problems among pregnant teens and adolescent mothers in rural areas, who require access to mental health services, support groups, financial assistance, housing support, and educational opportunities. However, providing support to pregnant teenagers in rural settings can be challenging due to limited access to mental health services, social stigma, financial restrictions, and poor transportation. Therefore, it is important to address these challenges and to provide adequate support to pregnant teenagers to promote positive mental health outcomes. To handle the mental health challenges of pregnant and parenting teenagers,, doctors, nurses, social workers, dietitians, psychologists, therapists and even personnel of the Department of Basic Education are needed to work as a multidisciplinary team [34].

### Limitations

The study was conducted in a selected surveillance area of Limpopo Province using the qualitative research approach. Thus, the findings cannot be generalised to other surveillance areas in different provinces. In terms of methods, mental problems were only self-reported without the use of the reporting scale for depression or stress. Furthermore, the sample of teenagers was based on those who were making consultations in selected primary healthcare settings.

## Conclusion

The study concluded that pregnant and parenting adolescents experience several challenges related to mental health problems during pregnancy and motherhood. Various factors contribute to mental problems among teenagers, including being a single parent, inadequate family support, and discrimination by the community. Although socioeconomic status and cultural factors also played a significant role in the type of support received, participants acknowledged the services provided by local social workers. Therefore, we recommend the implementation of integrated support programmes, which involve community membership and a multidisciplinary healthcare approach to address the financial, health and psychosocial needs of pregnant teenagers and mothers.

## Data Availability

Data generated or analysed during this study are included in this published article.

## Abbreviations

ANC: antenatal care
DSI: Department of Science and Innovation
LMICs: Low- and middle-income countries
PHRC: Population Health Research Centre
SA: South Africa
SAPRIN: South African Population Research Infrastructure Network
TREC: Turfloop Research Ethics Committee
